# Nurse-Led Psychological Intervention After Physical Traumas: A Randomized Controlled Trial

**DOI:** 10.14740/jocmr2082w

**Published:** 2015-03-01

**Authors:** Laila Skogstad, Erlend Hem, Leiv Sandvik, Oivind Ekeberg

**Affiliations:** a Department of Research and Development, Division of Critical Care, Oslo University Hospital, Ulleval, Oslo, Norway; b Department of Behavioral Sciences in Medicine, Faculty of Medicine, University of Oslo, Norway; c Unit of Biostatistics and Epidemiology, Oslo University Hospital, Oslo, Norway; d Department of Acute Medicine, Oslo University Hospital, Oslo, Norway

**Keywords:** Physically injured patients, Posttraumatic stress, Randomized controlled trial, Short-term psychological intervention

## Abstract

**Background:**

Emergency room nurses were trained to provide a short-term psychological intervention in physically injured patients with Impact of Event Scale (IES) scores > 20. The aims were to study the effects of the psychological intervention relative to usual care (UC).

**Methods:**

In a randomized controlled trial, psychological distress, daily functioning and the personality traits optimism/pessimism were compared with patients who received the UC. The interventions were provided 1 - 3 months after discharge.

**Results:**

The IES scores were significantly reduced in both groups at 3 months (intervention: 41.1 - 28.6, P < 0.001 vs. UC: 35.4 - 26.2, P < 0.001), but not significantly different between groups. Baseline IES score was a significant predictor of IES scores at 3 (β = 0.4, P < 0.05) and 12 months (β = 0.3, P < 0.05), whereas overall daily functioning at 3 months predicted IES scores at 12 months (β = -0.5, P < 0.001). Patients receiving intervention became significantly more optimistic during the year, and had an increase in overall daily functioning from 3 to 12 months (P < 0.001). Patients declining intervention were more pessimistic and had lower daily functioning. Patients who talked with nurses with more training in psychological processing had a larger reduction in IES symptoms at 3 months (β = -0.3, P = 0.081).

**Conclusion:**

The nurse-led intervention had a significant effect on optimism and overall daily functioning. Nurses may become a low-cost option to perform short-term psychological interventions with physically injured hospitalized patients.

## Introduction

Patients admitted to hospital after physical injury may develop psychological problems. The hospital staff primarily focuses on preventing somatic consequences [1]. Treatment of psychological responses is less prioritized [2], but patients treated in hospital may need psychological follow-up regardless of the severity of their injuries [3]. The prevalence of posttraumatic stress disorder (PTSD) 1 year following physical injury varies between 1% and 32% [4-6].

A review assessing effectiveness of psychosocial interventions following physical injury found heterogeneous studies, small sample sizes and high drop-out rates. No long-term effects were found, and some studies had detrimental effects [7]. Trauma-focused cognitive behavioral therapy (TF-CBT) seems to be the “gold standard” for early intervention [8]. There is evidence of long- [9] and short-term effects [10], but implementing CBT in an acute care setting is challenging because of clinical and logistical barriers [11].

New treatment approaches are studied, and the guidelines may change [12]. In a growing body of studies, the individual’s symptom profile is assessed and treated accordingly. The results differ; no differences in posttraumatic stress symptoms (PTSS) [13] or improvement in the severity of pain and psychological symptoms [14] were found. Two studies found less mental health problems [15, 16].

PTSD, chronic pain, psychiatric symptoms, and reduced functioning seem to be co-morbid after injury [17-19]. In addition, personality traits such as optimism/pessimism may influence recovery [20], severity [21] and cognitive functioning in patients with traumatic brain injury [22]. Negative life events may influence levels of optimism/pessimism [23]. Accordingly, optimism may not only be a personality trait but also be influenced by psychological interventions.

Nurses work closely with the patients [24, 25]. Breast cancer patients have been reporting more satisfaction with interventions with nurses compared to doctors [26]. Use of hospital nurses may be a less expensive solution (lower salaries, no rental for office, and low administration expenses). Nurses may help patients with the early processing of traumatic incidents, the hospital stay and return to normal functioning. They also know more about the injuries and somatic treatment than, e.g. psychologists, and the somatic physicians seldom have time for psychological follow-up. To our knowledge, this is the first report of nurses working in the emergency room leading psychological interventions to process posttraumatic distress symptoms.

The aims were to study the effects of a short-term, psychological intervention relative to usual care (UC): 1) primary outcome: level and predictors of PTSS at 3 and 12 months; 2) secondary outcome: level of daily functioning; and impact of personality traits optimism/pessimism.

## Materials and Methods

### Material

**Figure 1:**
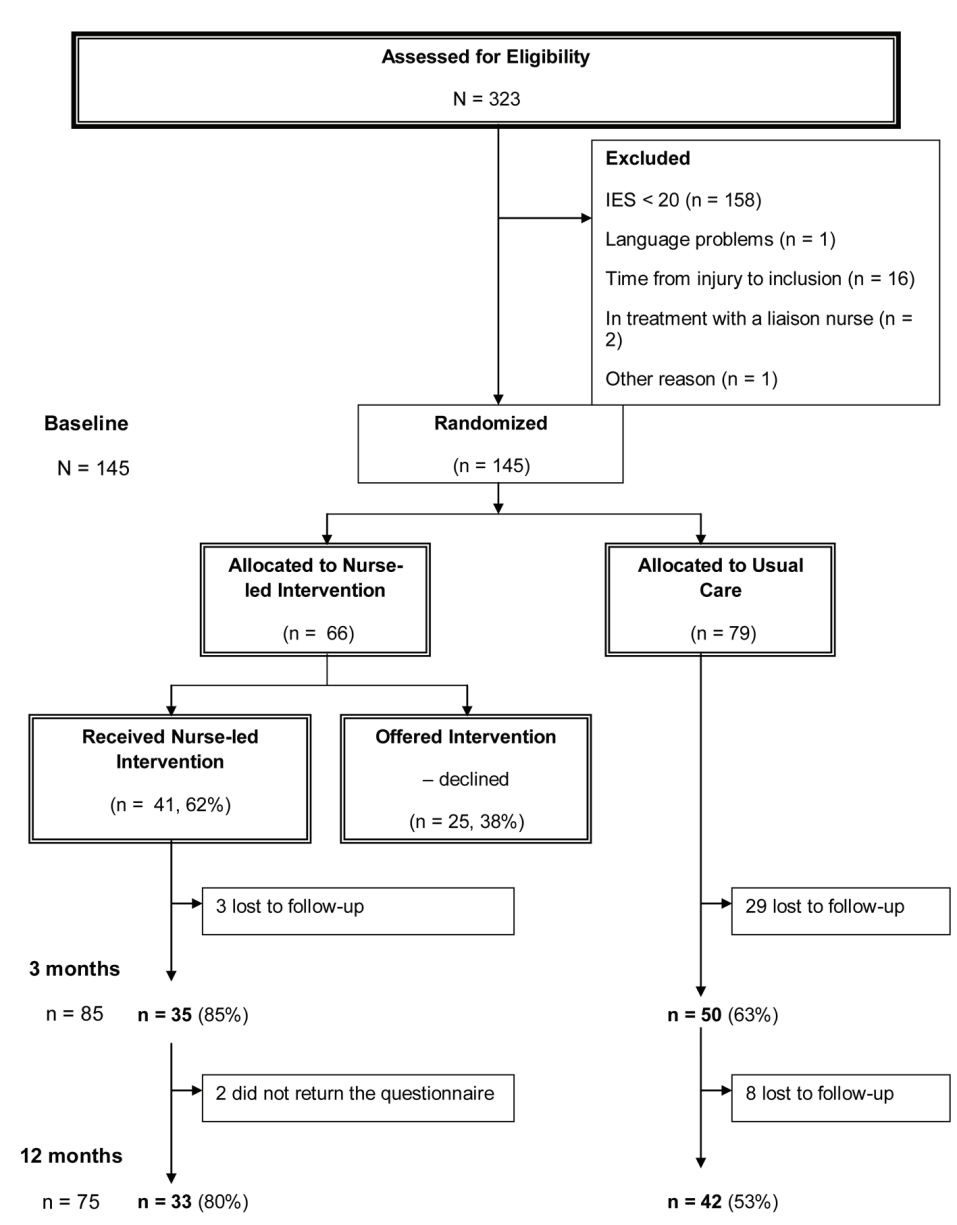
Flowchart for the nurse-led intervention vs. usual care.

This study was performed at Oslo University Hospital, Ulleval, Norway, a trauma referral center. Conscious adult patients (Glasgow coma scale (GCS) ≥ 11), 18 - 65 years old, with acute physical injuries (road traffic accidents, fall, violence and other injuries) were consecutively enrolled. A trauma-team was activated when the patient arrived at the hospital, and all patients were admitted for at least 6 h, some for days or weeks. A power analysis estimated a total of 160 patients, 80 in each group. At baseline, 323 conscious patients (GCS 14 - 15) responded (Fig. 1), of these 145 patients had an impact of event scale (IES) score ≥ 20 and were eligible for intervention. Patients were excluded if they had a stress score below clinical level (IES < 20, n = 158), lived more than 60 km from the hospital (too far for participating in the intervention), were incarcerated, unable to speak or read Norwegian, or had an unknown address, self-inflicted injuries, or serious psychiatric and/or substance abuse problems.

Before the allocation, 20 patients were excluded mainly because they returned their baseline questionnaires after 2.5 - 3 months, and thus it was not possible to conduct interventions before 3 months data collection. In addition, the inclusion took more time than expected, thus; the study had to finish somewhat earlier than intended.

Of the 66 patients assigned to the intervention, 41 (62.1%) agreed to participate, 85.4% responded at 3 months and 80.4% at 12 months. Twenty-five patients declined intervention. Of the 79 patients assigned to UC, 63.2% responded at 3 months and 53.2% at 12 months. Drop-outs between baseline and 3 months in the intervention group had lower educational level (P < 0.05). Drop-outs in the control group were more pessimistic at baseline (P < 0.05). Drop-outs in the group who declined intervention were younger (P < 0.05) and more lived alone (P < 0.05).

### Procedure

The list of patients admitted to the emergency room (ER) was checked each week. Written information was sent after the patients were discharged informing that a nurse would phone them approximately 1 week later. Self-report data were collected at baseline (1 - 2 months after the accident), and after the intervention at 3 months (3 - 4.5) and 12 months (12 - 14). A reminder was sent after 1 month.

Discharged patients answered and returned the baseline questionnaire before randomization. Randomization occurred in a 1:1 ratio according to a computer-generated random assignment sequence. Patients were offered 1 - 6 appointments.

### Treatment conditions

For both groups, nurses and doctors in the Division of Emergencies and Critical Care offered information, safety and care during the stay in hospital.

#### UC

No psychological treatment was administered from the hospital staff after discharge.

#### Nurse-led intervention

Five nurses were recruited from the emergency trauma team in the ER to perform the interventions. They had been working between 5 and 19 years, with a median work experience on the study ward (ER) of 5 years (range 3 - 15). All had supplementary education, but two had education/work experience relevant to perform psychological processing (education in communication skills and/or work experience from a psychiatric ward).

Preparation for the intervention included an 8-h training in issues such as posttraumatic stress symptoms and development of intervention skills. The nurses also participated in a 5-day course on crisis reactions, principles for processing feelings and adaptive responses [27].

Each intervention was preceded with a screening of the patients’ level of posttraumatic stress responses, as well as the level of functioning (work/studying, housework, leisure-time activities, friends and family). The intervention was based on cognitive behavioral methods. After the screening of posttraumatic stress responses, the patient was encouraged to make a narrative of the traumatic experience. Cognitive restructuring was performed focusing on dysfunctional behavior, restrictive thoughts, generalization and avoidant behavior. A checklist was used as guidance, but the narrative and the patient’s symptom profile was the basis for the intervention.

All interventions were performed between baseline and the 3 months data collection. The intervention was conducted in a separate room in the ER, and lasted about 60 min. Each nurse had appointments with five to nine patients, with a mean number of 2.5 (range 1 - 6). There were no significant correlations between the number of sessions and the effect of treatment.

### Measures

The IES measures posttraumatic stress on a six-point scale (from “never” to “a high degree” (total range 0 - 75) [28]). A cut-off of 19 is suggested as a clinically significant level of stress [28, 29]. IES total score refers to the sum of all 15 items. The subgroups intrusion and avoidance are also presented. In the present study, the internal consistency for IES was Cronbach’s α = 0.94.

The GCS measures level of consciousness from 3 to 15. A score of 3 reflects no response, and 15 reflects a normal level of consciousness [30].

The hospital anxiety and depression scale (HADS) measures states of anxiety and depression on a four-point (0 - 3) scale [31]. The sum of seven items provided the HADS-A (anxiety), and the sum of seven other items constituted the HADS-D (depression). The Cronbach’s α for anxiety was 0.88, and for depression was 0.86.

The life orientation test-revised (LOT-R) is a 10-item self-report scale (six target items and four fillers) that measures dispositional optimism/pessimism on a five-point scale from 0 (strongly disagree) to 4 (strongly agree) (range 0 - 24) [32]. A score above the median is considered optimism. The internal consistency for optimism was Cronbach’s α = 0.70 and for pessimism α = 0.76.

The abbreviated injury scale (AIS) classifies physical injuries by body region on a six-point scale from 1 (minor) to 6 (currently untreatable) [33]. The injury severity scale (ISS) is the sum of the squares of the three highest AIS scores in different body regions [34].

Previous psychiatric problems were assessed with six alternatives: 1) I have never had psychiatric problems; 2) I have had psychiatric problems, but have not consulted a general practitioner (GP); 3) I have consulted a GP for psychiatric problems; 4) I have been treated by a psychologist/psychiatrist; 5) I have consulted a psychiatric outpatient unit; and 6) I have been a psychiatric inpatient. Responses were collapsed into two categories: “no” (alternative 1) and “yes” (any other alternative).

#### Return to normal daily functioning

A liaison psychiatrist developed a questionnaire to measure five areas of daily functioning [35]: To what percentage are you back to normal functioning in the following areas: 1) work/studying, 2) housework, 3) leisure-time activity, 4) relationships and interactions with friends, and 5) relationships and interactions with family [34]. It has five response categories: 1) 0-20%, 2) 21-40%, 3) 41-60%, 4) 61-80%, and 5) 81-100%. A principal component analysis produced one factor (overall daily functioning), with a Cronbach’s α 0.91, that explained 75% of the variance.

#### Satisfaction with the intervention

Four questions measured satisfaction with the intervention. These questions were distributed only to those who had participated in the interventions: To what extent: 1) have you benefited from the interventions with the nurse; 2) has the nurse helped you process thoughts and feelings related to the accident; 3) did you experience support from the nurse; 4) has the duration of the intervention been satisfactory? Responses ranged from 1 (not at all) to 5 (to a very high degree). These questions were answered after the interventions were completed and again at 12 months.

### Data analyses

Data are presented as means with 95% confidence intervals (CIs), or percentages. Items with missing value were replaced by the mode of the others. Scores with more than one missing were not used, and only few scores could not be used. Accordingly, missing data have probably not biased the results significantly.

When appropriate, the demographic variables were dichotomized. Chi-square was used to compare proportions, independent sample *t*-tests and paired-sample *t*-tests compared means. Linear regression analysis was used to find predictors of posttraumatic stress at 3 and 12 months. A principal component factor analysis was used to estimate factor structure for the five questions regarding functioning. Statistical analyses were performed with SPSS software (version 18.0; SPSS, Chicago, IL, USA).

### Ethics

The Norwegian Data Inspectorate and the Regional Ethics Committee approved the study, and to use data from the Trauma Registry at the hospital. Approved trauma registrars at the Trauma Registry, calculated the AIS and ISS scores. This study was initiated before registration in a trial registry was mandatory, thus; it is not registered.

## Results

Patients in the intervention group were significantly older (M = 41.7 vs. M = 36.9, P < 0.05), and less often men (n =10 (28.6%) vs. 25 (50%), P < 0.05) than those in the UC group. A higher percentage in the intervention group reported college/university as their educational level (Table 1). There were no significantly differences between the groups regarding type of injury, injury severity, length of stay or discharge (Table 2).

**Table 1:** Background Variables for Physically Injured Patients in a Randomized Psychological Intervention Study

Variables	Nurse-led intervention (n = 35)	Usual care (n = 50)	P value intervention vs. usual care	Declined consultation (n = 17)
Age (years), mean (95% CI)	41.7 (37.2 - 46.3)	36.9 (33.0 - 40.7)	< 0.05*	41.8 (34.2 - 49.4)
Gender, male n (%)	10 (28.6)	25 (50.0)	< 0.05*	9 (52.9)
Level of education, n (%)			ns	
Primary and secondary school	13 (38.2)	30 (60.0)		8 (50.0)
College/university	21 (61.8)	20 (40.0)		8 (50.0)
Living status, n (%)				
Married/cohabitant - yes	19 (54.3)	24 (48.0)	ns	13 (76.5)
Custody of children - yes	15 (42.9)	20 (40.0)	ns	7 (43.8)
Working status, n (%)			ns	
Working/studying - yes	28 (80.0)	42 (84.0)		12 (70.6)
Previous psychiatric problems - yes	7 (20.6)	9 (18.4)	ns	3 (17.6)

Note: Patients with registrations at 3 months.

**Table 2:** Clinical Variables in Physically Injured Patients Measured During the Hospital Stay

Clinical variables	Nurse-led intervention (n = 35)	Usual care (n = 50)	P value intervention vs. usual care	Declined consultation (n = 17)
Type of injury, n (%)			ns	
Fall/other accident	7 (20.6)	9 (18.0)		7 (41.2)
Transport accident	26 (76.5)	38 (76.0)		10 (58.8)
Violence	1 (2.9)	3 (6.0)		0 (0)
Injury severity scale (ISS), mean (95% CI)			ns	
Minor/moderate (ISS score 1 - 8), n (%)	20 (58.8)	30 (62.5)		11 (64.7)
Serious (ISS score 9 - 15)	11 (32.4)	11 (22.9)		3 (17.6)
Severe (ISS score 16 - 24)	3 (8.8)	4 (8.3)		1 (5.9)
Critical (ISS score > 24)	0 (0)	3 (6.3)		2 (11.8)
Length of hospital stay, median (range)	2.0 (0 - 19)	2.0 (0 - 52)	ns	1.0 (0 - 14)
Discharged: other hospital/rehabilitation	6 (17.6)	15 (30.6)	ns	3 (18.8)

### Primary outcome

#### Posttraumatic stress symptoms

There was a significant reduction in IES scores in the intervention group, the UC group and the group that declined intervention from baseline to 3 months (Table 3). The mean IES score at baseline was significantly higher in the intervention group than in the UC group (M = 41.1 vs. M = 35.4, P < 0.05), but there were no significant differences between the groups at 3 and 12 months. The intrusion scores were significantly reduced from baseline to 12 months in both groups (mean difference: intervention 10.3 and UC 7.7, both P < 0.001). The avoidance scores were not significantly reduced in either of the groups.

**Table 3:** Psychological Distress and Optimism/Pessimism Measured the First Year Following a Physical Injury

Mean (95% CI)	Nurse-led intervention (n = 35)	Usual care (n = 50)	P value intervention vs. usual care	Declined consultation (n = 17)
Posttraumatic symptoms (IES)				
Baseline (1 month)	41.1 (36.9 - 45.4)	35.4 (32.2 - 38.6)	< 0.05*	38.4 (30.7 - 46.1)
3 months	28.6 (23.2 - 34.0)	26.2 (21.9 - 30.6)	ns	28.5 (20.1 - 36.9)
12 months	27.1 (20.7 - 33.4)	24.4 (18.0 - 30.9)	ns	25.3 (21.4 - 29.3)
P value within group: 1 - 3 months	< 0.001**	< 0.001**		< 0.05*
Anxiety (HADS-A)				
Baseline (1 month)	9.4 (8.2 - 10.6)	8.4 (7.1 - 9.8)	ns	8.3 (5.6 - 11.1)
3 months	7.3 (5.8 - 8.9)	7.3 (5.9 - 8.8)	ns	7.2 (4.4 - 10.1)
12 months	6.2 (4.5 - 7.8)	7.1 (5.3 - 8.7)	ns	6.8 (3.9 - 9.6)
P value within group: 1 - 3 months	< 0.05*	< 0.05*		ns
Depression (HADS-D)				
Baseline (1 month)	6.3 (5.0 - 7.6)	5.5 (4.3 - 8.7)	ns	6.1 (3.4 - 8.7)
3 months	4.6 (3.2 - 6.0)	4.8 (3.4 - 6.0)	ns	5.6 (3.3 - 7.8)
12 months	3.1 (1.8 - 4.4)	3.9 (2.3 - 5.5)	ns	3.7 (2.8 - 4.7)
P value within group: 1 - 3 months	< 0.05*	ns		ns
Optimism/pessimism (LOT-R)				
Baseline (1 month)	15.6 (14.0 - 17.1)	14.9 (13.6 - 16.3)	ns	11.9 (9.7 - 14.0)
12 months	17.3 (15.4 - 19.2)	14.8 (12.7 - 16.9)	0.068	15.3 (11.8 - 18.8)
P value within group: 1 - 12 months	< 0.05*	ns		ns

Note: Patients with registrations at 3 months. *P < 0.05, **P < 0.001.

#### Predictors of posttraumatic stress at 3 and 12 months

Posttraumatic stress (IES) at baseline was a significant predictor (β = 0.4, P < 0.05) of posttraumatic stress at 3 months (Table 4). There was no statistically significant effect of the nurse-led intervention. The two nurses with more psychological training, however, had a clinically important independent effect (P = 0.081, Table 4) with baseline IES scores of M = 43.4, and M = 23.0 at 3 months. The corresponding figures for the nurses with less psychological training were M = 40.1 and M = 30.7, respectively.

**Table 4:** Predictors of Posttraumatic Stress in Physically Injured Patients 3 and 12 Months After Psychological Nurse-Led Intervention (Linear Regression Analyses)

	Unadjusted (enter) variables				Adjusted (multivariable) (backward) variables			
	β	95% CI	*t*	Sign.	β	95% CI	*t*	Sign.
3 months								
Age: continuous	0.3	-0.2-0.3	0.3	ns				
Gender: 1 = man, 2 = woman	-0.1	-12.7 - -0.6	-1.9	ns				
IES baseline (sum score)	0.4	0.3 - 0.8	4.6	< 0.001**	0.4	0.02 - 0.8	2.2	< 0.05*
LOT baseline (sum score)	-0.9	-1.6 - -0.3	-2.8	< 0.05*				
Nurse-led intervention: yes/no	-0.8	-9.1-4.4	-0.7	ns				
Psych trained nurses: 1 = no, 2 = yes^a^	-0.2	-18.4-3.1	-1.4	ns	-0.3	-19.4-1.2	-1.8	0.081
12 months								
Age: continuous	0.04	-0.3-0.4	0.3	ns				
Gender: 1 = man, 2 = woman	-0.1	-11.4-7.2	-0.4	ns				
IES baseline (sum score)	0.4	0.2 - 0.9	3.0	< 0.05*	0.3	0.1 - 0.8	2.9	< 0.05*
LOT baseline (sum score)	-1.2	-2.0 - -0.4	-2.9	< 0.05*				
Nurse-led intervention: yes/no	-0.1	-11.5-6.2	-0.6	ns				
Psych trained nurses: 1 = no, 2 = yes^a^	-0.2	-21.9-4.3	-1.4	ns				
Functioning at 3 months (sum score)	-0.5	-1.8-0.6	-4.1	< 0.001**	-0.5	-1.8-0.7	-4.4	< 0.001**

Dependent variable: IES sum score at 3 and 12 months. ^a^With more relevant training for psychological processing. Note: Patients in the intervention and usual care groups. *P < 0.05, **P < 0.001.

Posttraumatic stress at baseline was also a significant predictor of posttraumatic stress at 12 months (β = 0.3, P < 0.05), as was the overall daily functioning sum score at 3 months (β = -0.5, P < 0.001).

#### Anxiety and depression

There was a significant reduction in HADS anxiety from baseline to 3 months in both the intervention group (M = 9.4 vs. M = 7.3, P < 0.05) and the UC group (M = 8.4 vs. M = 7.3, P < 0.05), but not among those who declined intervention (Table 3). For HADS depression, a significant change was only detected in the intervention group (M = 6.3 vs. M = 4.6, P < 0.05). The multivariate analyses, however, showed no significant independent effect of the nurse-led intervention.

### Secondary outcome

#### Return to normal daily functioning

Work/studying and leisure-time activities improved most from 3 to 12 months. A significant increase in overall functioning was only found in the intervention group between 3 and 12 months (mean difference: intervention 2.9, P < 0.001 and UC 1.9, ns). However, there was no independent effect of the intervention on daily functioning.

#### Life orientation traits

Patients in the intervention group became more optimistic (M = 15.6 vs. M = 17.3, P < 0.05), with no significant change in the UC group. Those who declined participation were significantly more pessimistic compared with the intervention group (M = 11.9 vs. M = 15.6, P < 0.05). There was no independent effect of LOT-R on the IES or HADS scores.

#### Satisfaction with the intervention

The patients had high satisfaction scores for all four items. 1) Have you benefited from the interventions; 2) Has the nurse helped you process thoughts and feelings; 3) Did you experience support; 4) Has the duration of the intervention been satisfactory, with means ranging from 3.8 to 4.5 (scale 1 - 5). Item 4) showed the same level at 3 and 12 months (M = 3.9); all others increased insignificantly.

## Discussion

The IES scores decreased significantly in both groups, even though there were no significant differences between groups. Patients in the intervention group became significantly more optimistic and achieved a significantly better daily functioning.

Patients in the intervention group also had a significant decrease in depressive symptoms. In addition, they reported a high degree of satisfaction with the intervention.

Patients declining intervention had lower baseline IES and anxiety scores compared to those receiving intervention. Accordingly, they may be less motivated for treatment. In the general Norwegian population (women and men aged 30 - 49 years), HADS anxiety scores of 4.2 - 4.6 and HADS depression 2.7 - 3.6 were found [36]. All groups in the present study scored higher than the general population, except for depression at 12 months.

In one study using early CBT, no positive impact on anxiety and depression symptoms, but a modest reduction in PTSD score in the intervention group were found [9]. To be included, a high IES (> 35) and HADS score (> 15 in each sub-scale) were required. Early CBT has also been performed on patients with acute PTSD [10] without an effect on PTSD, anxiety and depression. Most studies of early intervention performed in hospitals have not found significant improvement in posttraumatic stress symptoms in the intervention group, but there have been interesting findings on other measures [13-16, 37].

Most researchers using the LOT-R emphasize that it measures a personality trait rather than a state. Patients receiving the intervention became significantly more optimistic compared to the UC group and those declining intervention. This may indicate that the LOT-R measures both state and trait characteristics, and that a psychological intervention may promote optimism. Only patients in the intervention group achieved significantly better overall daily functioning. The nurses had focus on functioning (work-related and social/psychological) as well as posttraumatic responses. This may have helped the intervention patients to be aware of dysfunctional or avoidant behavior and restrictive thoughts. An increase in optimism and overall functioning in the intervention group may be supported in one study of patients with moderate-to-severe traumatic brain injury, where higher levels of dispositional optimism was related to better psychological functioning [22].

The patients were satisfied with the intervention. Their degree of satisfaction was equivalent with ICU patients’ satisfaction with communication in our hospital [38].

### Training

The numbers of sessions and patients were limited and thus, one should be careful with the interpretation. The patients might have benefited from more sessions even though they were satisfied with the duration. Further studies may consider more pre-intervention training for the nurses, a larger number of patients for each nurse, and possibly more than two or three appointments. To our knowledge, this is the first nurse-led psychological intervention with trauma patients. We consider this low-threshold and low-cost intervention to be of interest in further investigations.

### Strengths and limitations

Inclusion was restricted to patients living less than 60 km from the hospital. This may have increased the generalizability because those who are willing to travel longer distances could introduce bias to the sample. It is likely that unconscious patients might have an even greater need for interventions. The originality of the study is considered a strength. Some patients are reluctant to consult psychologists or psychiatrists, and it may be less stigmatizing to attend a routine follow-up with a nurse. We consider the results to be generalizable to other major trauma centers in Western Europe.

The level of PTD was significantly higher in the intervention group at baseline, which was controlled for in the regression analyses. This difference may be a possible limitation. Those with higher scores might have been more motivated for participating, and high baseline stress score is probably more prone to a regression to the mean. Based on the higher baseline stress score in the intervention group, one should expect somewhat higher scores also at the follow-ups. There were 80.5% in the intervention group and 53.2% in the UC group who completed the assessments. The adherence to assessment is considered satisfactory for the intervention group, but less satisfactory for the UC group. Patients who were interested in the intervention were more often women and had higher education. The same pattern was found among the completers in both groups. The lower response rate in the UC group may reflect a lower interest in participating in a study without treatment. The pattern of drop-out indicates that the findings can be generalized especially to women and to patients with higher education.

Our moderate sample size is larger than some studies [2], or comparable to others [13, 16].

### Implications

Including only patients at PTSD level might have improved the results, and an assessment of the motivation for treatment might identify patients with greater needs. The current intervention was initiated approximately 1 month after discharge, but many patients develop symptoms later [39, 40], and professionals should also address these patients.

Nurses may play an essential role in screening and early intervention. If the patients still have distress symptoms after four or five interventions, the patient can be referred to further treatment.

### Conclusions

Even though the nurse-led intervention failed to reduce posttraumatic stress symptoms relative to the UC group, the patients in the intervention group became more optimistic, increased the level of daily functioning and reported less depressive symptoms. In addition, they reported a high degree of satisfaction with the intervention. Nurses may become a low-cost option to perform short-term psychological interventions with physically injured hospitalized patients.
